# Progressive Resistance Exercises plus Manual Therapy Is Effective in Improving Isometric Strength in Overhead Athletes with Shoulder Impingement Syndrome: A Randomized Controlled Trial

**DOI:** 10.1155/2021/9945775

**Published:** 2021-06-30

**Authors:** Saurabh Sharma, Amer K. Ghrouz, M. Ejaz Hussain, Shalini Sharma, Mosab Aldabbas, Sumbul Ansari

**Affiliations:** ^1^Centre for Physiotherapy and Rehabilitation Sciences, Jamia Millia Islamia, New Delhi, India; ^2^Department of Applied Medical Sciences, Faculty of Medicine and Health Sciences, An-Najah National University, Nablus, State of Palestine; ^3^Department of Medicine, Universitat Autònoma de Barcelona, Barcelona, Spain; ^4^Enrich Physio Clinics, Melbourne, Victoria, Australia

## Abstract

Reduction in isometric strength of the scapulohumeral muscles is a commonly seen impairment in overhead athletes afflicted with shoulder impingement syndrome (SIS). The purpose of this study was to compare the effects of two different treatment programs: progressive resistance exercises plus manual therapy (PRE plus MT) and motor control exercises (MCE), on isometric strength of upper trapezius (UT), middle trapezius (MTr), lower trapezius (LT), serratus anterior (SA), supraspinatus (Supr.), anterior deltoid (A.D), and latissimus dorsi (LD). 80 male university-level overhead athletes clinically diagnosed with SIS were randomly allocated into either of the two groups: PRE plus MT and MCE group. Athletes in the PRE plus MT group underwent graduated exercises with resistance elastic band, stretching exercises, and mobilization of the thoracic and shoulder joints. MCE group was submitted to motor control exercises in varied planar positions. Athletes in both groups underwent management 3 times a week for 8 weeks. Isometric strength of UT, MTr, LT, Supr, A.D, SA, and LD was measured at three-time points: baseline, 4th week, and 8th week. Relative to baseline, both interventions were found to be effective in increasing and optimizing the isometric strength of muscles (*p* < 0.05) except for supraspinatus in the MCE group (*p* > 0.05). However, athletes in PRE plus MT group presented a more pronounced increase in isometric strength than those in the MCE group. Between groups analysis found the largest isometric strength improvement in PRE plus MT group for A.D, followed by Supr. and UT muscles (*p* < 0.05; effect size: 0.39 to 0.40). The study concluded that compared to MCE, PRE plus MT provides greater improvement in the isometric strength of scapulohumeral muscles.

## 1. Introduction

Shoulder impingement syndrome (SIS) is a common musculoskeletal condition seen in overhead athletes, and it affects a large number of athletes at some point during their career [1]. Overhead athlete is referred as to one who regularly uses his arm above 90 degrees during play. SIS is a term that describes a condition where the subacromial structures are compressed between the humeral head and the coracoacromial arch causing a myriad of signs and symptoms [2].

The reduction in subacromial space can also occur due to mechanistic effects and improper scapular fixation resulting in compromise of the subacromial space and its structures [3]. The most common clinical features include pain, limited range of motion, and decreased strength in the arm. Reduction in isometric strength of shoulder external rotation and abduction are the two most cardinal features of SIS. The reduction in isometric strength could be firstly due to muscle deconditioning following SIS onset and secondly related to deficits in motor control of the muscles [4, 5]. Lack of motor control changes the muscle activation levels and decreases the coordination between the glenohumeral and scapulothoracic muscles during the overhead elevation motion, and eventually, it contributes to the reduction of isometric strength [5]. The muscles mainly affected by isometric strength reduction are upper trapezius (UT), middle trapezius (MTr), lower trapezius (LT), serratus anterior (SA), supraspinatus (Supr), anterior deltoid (AD), and latissimus dorsi (LD) [5]. Isometric strength changes up to 33% and 29% in shoulder external rotation and shoulder abduction, respectively, have been reported in studies [6]. In comparison, the deficits in protraction and retraction have been reported to be 8% and 18%, respectively [6]. The isometric strength improvement could be considered as a prognostic marker in SIS as it is markedly reduced in most cases. There is a paucity of evidence due to a limited number of studies that have examined isometric strength improvement as their primary outcome.

SIS contributes 27% of the total shoulder injury burden in overhead athletes [7]. The prevalence of shoulder injuries is high in athletes because of repetitive overhead use and inadequate conditioning in the early phase of the season [7]. Due to multiple etiological and pathomechanical pathways of SIS, a wide range of treatment strategies has been proposed for the management of SIS with each strategy aiming to target a particular mechanical pathway [8, 9]. Isometric strength improvement can occur by two mechanisms. First is the neuromuscular adaptations resulting from increased motor unit recruitment to hyperplasia of muscle fibres occurring due to exercise therapy [10]. Second is the reduction in alpha motor neuron inhibition due to manual therapy by reducing pain level and hypomobility in the spinal and related joints.

Both progressive resistance exercises (PRE) and motor control exercises (MCE) are the most frequently and commonly used conservative techniques. In the systematic reviews of clinical trials, it was reported that PRE had a positive impact on the outcomes of SIS (pain, strength, range of motion, and function) [11, 12]. A few studies and systematic reviews have concluded that exercise therapy is equivalent or even better than surgical procedures as similar outcomes can be achieved by exercise therapy alone [13, 14]. Both PRE and MCE have been shown promising results in the management of SIS [15–17]. PRE exercises work on the principle of producing physiologic adaptations by activating a greater number of satellite cells [4]. In contrast, the MCE changes the altered muscle activation levels and corrects the control and coordination of the glenohumeral and scapulothoracic joint during the overhead elevation motion [5]. Maitland mobilization is one of the widely used manual therapy (MT) techniques for the management of musculoskeletal dysfunctions [18]. Maitland's concept of mobilization is an evolved system of assessment, clinical reasoning, and hypothesis framing and treatment. The assessment includes a detailed patient interview followed by an examination of the physiologic and accessory motion (roll, spin, and glide). The findings of the interview and examination are corroborated with each other, and then, the therapist uses the findings as a treatment tool [19]. The oscillatory technique of mobilization is used in this concept for vertebral and peripheral joints to treat pain, hypomobility, and increase capsular extensibility of mechanical nature. Studies examining manual therapy techniques in isolation have also reported that manual therapy is effective in relieving pain and hypomobility and improving the strength of muscles [20, 21]. There have also been largely inconclusive results of studies and systematic reviews on the effects of the combination of resistance exercise and manual therapy in patients with SIS. Some studies concluded that manual therapy should be used as an additive component of resistance exercise, while authors in other studies could not give a strong recommendation for use of a combination of resistance exercise and manual therapy [15, 22–25]. The main reason for not being able to draw strong recommendations is that most of the trials to date have included heterogeneous samples, poor exercise prescription in terms of frequency, time, type, and intensity and usually studied the effects of resistance exercise and manual therapy in isolation. Regarding the abovementioned isometric strength impairment, PRE, MT, and MCE seem to scientifically form an essential and integral component of the treatment regime of athletes diagnosed with SIS. As per recommendations in earlier research, in this study, PRE was combined with MT so that restored mobility of the joint is retained with the help of exercises [26]. Because of evidence obtained from the literature review, the hypothesis that can be derived from findings is that altered isometric strength of the scapulothoracic muscles affects the shoulder girdle movements and plays a major role in the sustenance of the SIS. Therefore, an improvement in the isometric strength possibly could help in mitigating the symptoms in overhead athletes suffering from SIS.

However, to the best of our knowledge, there is a dearth of studies that have investigated the effectiveness of PRE plus MT and MCE; therefore, it seems rationale and imperative to study these intervention programs in overhead athletes affected by SIS. The present study was aimed to compare the effects of two different treatment interventions: PRE plus MT versus MCE on isometric strength of scapulothoracic muscles (UT, MTr, LT, SA, Supr, AD, and LD) in overhead athletes with SIS. We hypothesized that both treatment interventions (PRE plus MT and MCE) will result in improvement of isometric strength of shoulder and scapular muscles.

## 2. Material and Methods

### 2.1. Participants

Athletes were recruited in the study as per the following criteria: (i) 17-35 years of age; (ii) male overhead athletes playing competitive sports like volleyball, tennis, baseball, cricket, swimming, badminton, and basketball for at least 6 hours a week; (iii) shoulder impingement symptoms duration of ≥1 month; (iv) pain rating on visual analog scale (VAS) of less than or equal to 7/10 (during elevation activity); (v) athletes agreeing to participate for the entire duration of treatment; and (vi) athletes were recruited on fulfilling a minimum of 2 out of 5 diagnostic criteria for SIS. The following diagnostic criteria were used for clinically diagnosing SIS criteria but not based on the last two criteria alone [27]: (a) Positive Neer's sign, (b) Positive Hawkins sign, (c) Positive Jobe's sign, (d) Positive Apprehension test, and (e) Positive relocation test. Subjects were excluded when they have any of the following: prior shoulder dislocation in the same or opposite shoulder, athletes with bilateral shoulder pain, acromioclavicular (AC) joint pathology, cervical spine radiculopathy, athletes currently on medicines like NSAIDs, prior shoulder surgery on the symptomatic side, or positive drop arm test for full-thickness rotator cuff tear.

### 2.2. Study Design

The present study was a randomized controlled trial with a two-arm parallel repeated measure design and was conducted at the physiotherapy clinic of the university (Jamia Millia Islamia-A Central University).

### 2.3. Ethical Consideration

All procedures performed in the study were in accordance with the ethical standards of the institutional/or national research committee (Institutional Ethics Committee, Jamia Millia Islamia (vide no 07/04/JMI/IEC) and with the 1964 Helsinki declaration and its late amendments or comparable ethical standards. The trial was registered at the central trial registry vide no. CTRI/2018/05/013892.

### 2.4. Sample Size

The sample size for the study was determined by the use of the statistical program G∗ power Software (version 3.1.9.4; Henrich-Heine-Universitat Dusseldorf, Germany) [28]. The calculations were based on the assumption of finding a 30 percent difference in primary outcome measure, i.e., isometric strength of internal rotator, at an alpha level of 0.05, Cohen *d* = 0.60 effect size and power (1 − *β*) of 0.80 [15]. This generated a sample size of 34 subjects in each group. After accounting for a dropout of 15%, a total of 78 subjects were found to be necessary to find meaningful differences between groups.

### 2.5. Procedures

**Figure 1:**
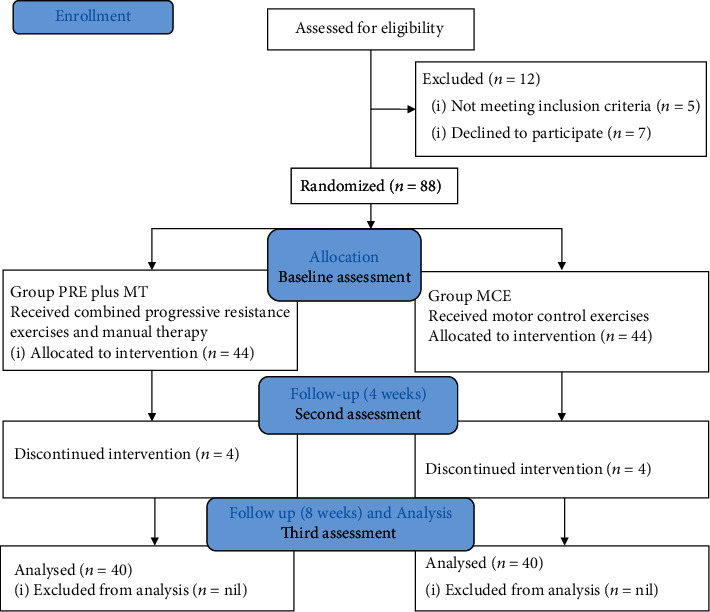
Details of the study protocol.

The recruitment was done through the screening process of athletes with shoulder pain who came to the clinic after reading flyers posted online on the university website. The athletes were physically examined as per the eligibility criteria, and those who agreed to participate were recruited for the study. The athletes were randomly allocated to one of the two groups: PRE plus MT group and MCE group, by using a computerized random number generator. The total duration of the study was 8 weeks. The baseline measurement of isometric strength of muscles (UT, MTr, LT, SA, Supr, AD, and LD) was recorded on the day of the recruitment. The entire process of examination and recording baseline measurements took around 30 minutes on average. The recordings in the study were taken at three-time points, i.e., baseline, 4th week of intervention, and 8th week of intervention for isometric strength of the muscles. The study protocol is given in Figure 1.

### 2.6. Measurements

#### 2.6.1. Isometric Strength Measurement

For the evaluation of isometric strength of muscles, the Lafayette® handheld dynamometer (HHD) Model-01165 (Lafayette Instrument Company, Lafayette IN, USA, 2013) system was utilized. It is a microprocessor-controlled handheld instrument for the quantitative assessment of isometric muscle strength. The measurement range of the instrument is up to 1335 N. Isometric muscle strength is the ability of the subject to hold a sustained contraction against an unyielding resistance for a specified duration of time. In isometric contraction, there is no movement of the joint but due to contraction; tension is generated in the muscle. The isometric strength was assessed by applying counter force with the HHD, opposite to the athlete's direction of the force. HHD is a reliable instrument for measuring muscle strength [29]. The athlete was required to perform maximal contraction of the muscle while the examiner applied counter-force to achieve isometric contraction. A familiarization session was done before actual testing. The athlete performed three trials (maximal strength) such that the average of the maximal strength recordings was used for analysis (sum of maximal strength trials/no. of repetitions). One minute of rest was provided between each trial. The maximal isometric strength was recorded by the device during the test in Newton. The isometric measurement was measured for UT, MTr, LT, SA, Supr, AD, and LD. The isometric strength was measured by utilizing the following positioning as mentioned in Table 1 [29].

### 2.7. Interventions

Athletes were randomly assigned to one of the two intervention groups: PRE plus MT group and MCE group for 8 weeks protocol. The intervention in both groups was performed by a physiotherapist having clinical experience of 10+ years after postgraduate education and also having add-on requisite manual therapy certifications. The pre- and postassessment were performed by the different assessors (physiotherapist) who were blinded to group allocation and treatment protocols.

#### 2.7.1. Progressive Resistance Exercises plus Manual Therapy (PRE plus MT) Group

Athletes in the PRE plus MT group underwent an amalgamated protocol of resistance exercise and manual therapy [24, 30]. The protocol aimed to strengthen shoulder and scapular muscles, regain range of motion (ROM) of the shoulder quadrant joints, and stretch the shortened muscles. The total protocol duration was of 8 weeks. The strengthening component of the protocol was to be performed 3 times a week, while the ROM exercises were to be done on daily basis for 10 repetitions (reps). Stretching exercise also comprised an important component of the protocol, and 5 repetitions daily withhold of 30 seconds for each repetition were performed by the athletes. The program began with ROM exercises that included shoulder retraction (athletes actively performed shoulder external rotation by keeping elbow in a flexed position), pendulum exercise (athletes performed swinging movement of shoulder joint in a clockwise and counter-clockwise direction for one minute each), an active training of scapula muscles (athletes performed scapular pullbacks while keeping the arms by the side), active-assisted exercises with the cane (athletes performed medial and lateral rotations, flexion, and diagonal elevation by holding a cane with both hands and applied force primarily from the normal side), and posture exercises (athletes were taught to self-correct their abnormal hiking of shoulder while performing active shoulder elevation in front of a mirror). Participants were also educated to perform stretching of the anterior shoulder (athletes while placing their forearms and hand on the wall, stood at an arms distance and then the athlete leaned forward) and posterior shoulder capsule (athletes stood against the wall and while anchoring the affected side scapula brought the affected shoulder into cross-body adduction in such a manner that stretch was felt in the back of the shoulder).

Maitland manual therapy grades (nonthrust) I to IV were used for treatment. A total of 12 MT sessions were administered over a period of 8 weeks [19, 31]. The athlete was in the prone position and the physiotherapist performed the thoracic PA (posteroanterior) glides by keeping the elbow straight and used the pisiform bone to apply the PA glide on the thoracic spinous process. To apply precise mobilization, the physiotherapist hooked the fifth finger of the above hand with the index finger of the bottom hand. Glenohumeral posterior and inferior glide mobilizations were performed by placing the athlete in a supine position with a towel under the scapula. The physiotherapist placed one hand over the humeral head (for posterior glide) and lateral to the acromion (for inferior glide), while the other hand supported the elbow and then a glide in posterior and inferior direction was applied to the humeral head in different elevated positions of below and above 90 degrees. The stability of the shoulder complex was improved by performing strengthening exercises. The duration of phase 1 progressive resistance exercises (PRE) lasted up to two weeks from the inception of the exercises. The exercises were performed with an elastic resistance band by moving in the opposite direction of band fixation (held by the examiner). The resistance exercises comprised of shoulder lateral rotation and medial rotation in a neutral position, scapular retraction (athlete held the resistance band with both hands and then pulled it outwards followed by slow release while keeping the elbow inflexed position), and resisted scapular protraction (bodyweight secured band was pushed towards the ceiling such that the shoulder blades were lifted off the table), while the elbow was kept extended, scapular retraction with chin tuck-in.

The progression pattern of the resistance exercise regime in phase 1 comprised graduating the repetition counts from starting 2 sets of 10 reps to 3 sets of 10 reps. The starting resistance of the elastic resistance band (colour code) was selected on the criteria of repetition maximum, i.e., resistance band that permitted an athlete to complete 8 to12 repetitions per set to the point of fatigue [32]. On every 4^th^ day of this phase, progression was made by changing the colour of the resistance band (different colours denote varying resistance) [4] [Table 2].

The duration of phase 2 progressive resistance exercises (PRE) was also 2 weeks. The exercises were performed with the elastic resistance band by moving in the opposite direction (followed by slow descent) of band fixation (held by the examiner), namely, shoulder elevation and flexion up to 90°, shoulder lateral and internal rotation in shoulder abducted and elbow flexed position of 45°-90°, shoulder resisted extension from 45° flexed position, quadruped push-up plus “camel” (athlete was in all four positions and then upper trunk slouching was performed by pushing downwards followed by gradually release), and scapular “T” and “Y” exercise (athlete was in prone lying with the shoulder in abducted and thumbs turned up position, following this athlete raised the arms towards the ceiling and/or raised diagonally up, while contracting scapula together towards spine). At the end of the 4^th^ week of progression of the resistance, the program consisted of an increase in the repetition counts from the initial 2 sets of 10 repetitions to 3 sets of 10 repetitions. The progression of the band was done every week in this phase (Table 2).

From the 5^th^ to 8^th^ week of the therapeutic program, phase 3 progressive resistance exercises (PRE) were performed. With the addition of two new exercises, other exercises remained the same as in phase 2. The exercises comprised of chair press (athlete while in sitting position tried to lift the buttocks off the chair) and “protraction plank” (athlete while in prone plank posture with the thoracic spine in an extended position (slouched) pushed downward through the forearm producing upper trunk spinal flexion position and after that slowly returned to starting position). The progression was made by increasing the repetition count at the end of the 8th week [4] (Table 2).

#### 2.7.2. Motor Control Exercise (MCE) Group

The motor control exercises mainly composed the MCE group. Athletes in the MCE group were submitted to a group of six free exercises for the upper quadrant region. The range of exercises consisted of shoulder abduction in the frontal plane (athlete while in the standing position, raised the arms in the frontal plane to perform sideways elevation), shoulder retraction (athlete brought the two shoulder blades together during standing position by laterally rotating the arms and held for five seconds), neck retraction (athlete drew his neck in backward position as if trying to touch chin to the throat), shoulder shrugging (athlete while in the standing position, elevated both the shoulder and brought them closer to the ear for a duration of five seconds), stretching of upper trapezius (athlete while in the chair sitting position, pulled one of the shoulders back by holding onto the chair while the other hand grasped the head region and pulled the neck in the opposite direction toward the armpit and held it for a duration of 20 seconds), and pectoralis major (athlete was in lying position, the arm was raised to 125°for duration of 20 seconds and then gradually go down towards the floor) [13] (Table 3).

### 2.8. Analysis

Data analysis was conducted with the SPSS software (version 21.0, SPSS Inc., Chicago, IL). The statistical analysis was framed to analyze the within-subject and between-subject effects over all the time points. The data is presented as mean ± SD, and the normality of the data was assessed with the Shapiro Wilk test. The baseline demographic and descriptive characteristics of the two intervention groups were assessed through an independent sample *t*-test. Mean change in isometric strength of muscles was also calculated between baseline to midtreatment (4th week) and baseline to 8 weeks of treatment. Split-plot repeated measure ANOVA (2x 3 models) was conducted to find the effect of time, group, and time × group effect on the isometric muscle strength (UT, MTr., LT, SA, A.D, Supr., and LD). Mauchly's test was used as a reference, and Green-Geisser correction was applied if sphericity was violated. Effect size (ES) was used as an indicator of the treatment effect, which was defined as small (0.20), moderate (0.50), and large (0.80) [33]. Percentage change was calculated from baseline to the 8th week by using the formula: pre − post/pre∗100 [34]. For the present study, the level of significance was considered as *p* < 0.05.

## 3. Results

**Figure 2:**
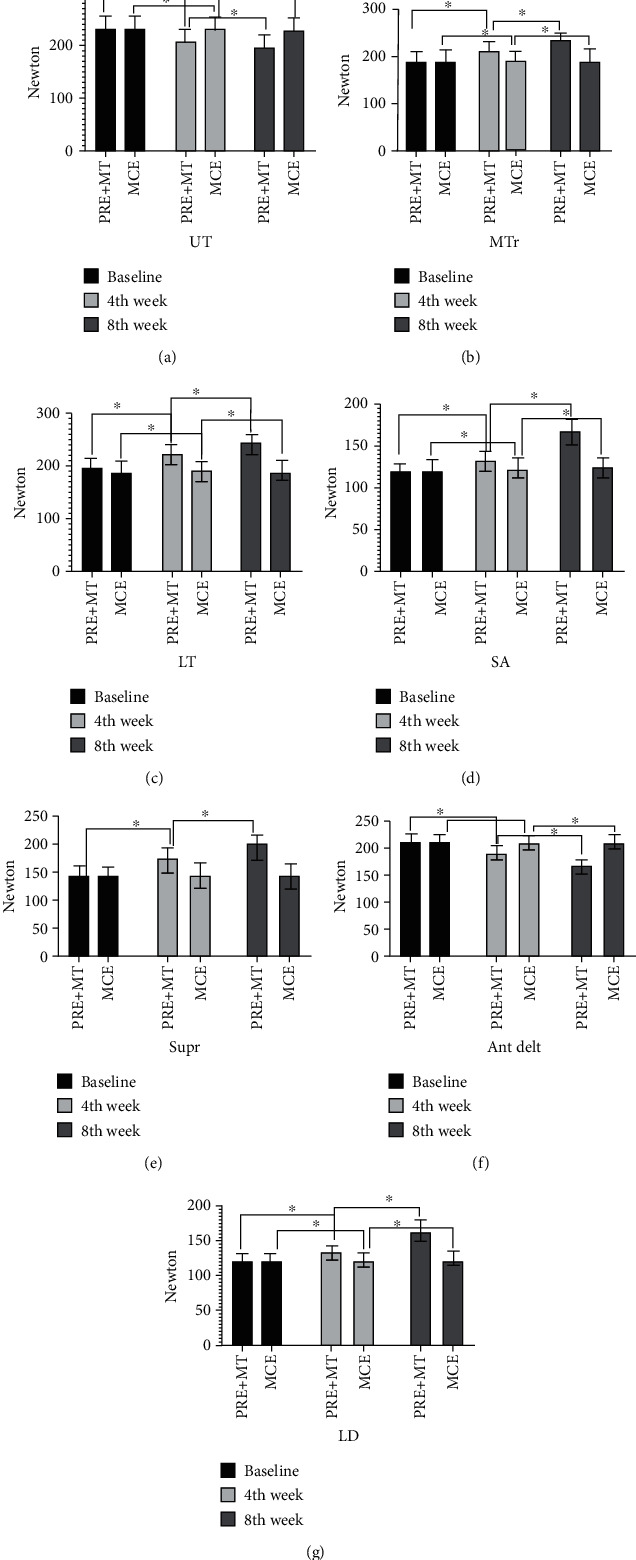
(a) UT isometric strength for the PRE plus MT and MCE group at baseline, 4^th^ and 8^th^ week. Data are presented as mean ± SD. ^∗^ Significant difference for *p* < 0.05. (b) MTr isometric strength for the PRE plus MT and MCE group at baseline, 4^th^ and 8^th^ week. Data are presented as mean ± SD. ^∗^ Significant difference for *p* < 0.05. (c) LT isometric strength for the PRE plus MT and MCE group at baseline, 4^th^ and 8^th^ week. Data are presented as mean ± SD. ^∗^ Significant difference for *p* < 0.05. (d) SA isometric strength for the PRE plus MT and MCE group at baseline, 4^th^ and 8^th^ week. Data are presented as mean ± SD. ^∗^ Significant difference for *p* < 0.05. (e) Supr. Isometric strength for the PRE plus MT and MCE group at baseline, 4^th^ and 8^th^ week. Data are presented as mean ± SD. ^∗^ Significant difference *p* < 0.05. (f) Ant Delt. Isometric strength for the PRE plus MT and MCE group at baseline, 4^th^ and 8^th^ week. Data are presented as mean ± SD. ^∗^ Significant difference for *p* < 0.05. (g) LD isometric strength for the PRE plus MT and MCE group at baseline, 4^th^ and 8^th^ week. Data are presented as mean ± SD. ^∗^ Significant difference for *p* < 0.05.

A total of 88 overhead athletes with SIS were recruited for the study. There was a dropout of four athletes from each group during the intervention. A total of eighty athletes (PRE plus MT group mean age: 21.30 ± 2.10 years, MCE group mean age: 21.80 ± 2.80 years) clinically diagnosed with SIS agreed to participate and completed the study. The demographic and descriptive measurements of the isometric strength outcome of the muscles in the two groups were not found to be significant at the baseline level (*p* < 0.05) (Table 4). The mean changes in the isometric strength measured from baseline to the 4th week of intervention were found significant (*p* < 0.05) for all muscles in both the groups except for supraspinatus muscle in the MCE group (Table 5, Figure 2). This statistically significant improvement of isometric strength was found for all the muscles in both groups when recordings were again done postintervention at the end of the 8th (*p* < 0.05). MCE group did not show improvement in supraspinatus isometric (*p* < 0.05) (Table 5, Figure 2). The mean change comparison of groups before and after treatments revealed that both groups presented an increase in isometric strength. However, the PRE plus MT group presented a significantly higher increase as per the effect size value in isometric strength of muscles than those in the MCE group.

The percentage change in PRE plus MT and MCE group was statistically significant (*p* < 0.05) and in the range of 21.48 to 41.78% and was 0.42 to 2.08%, respectively. Only supraspinatus muscle in the MCE group did not have significant change (*p* < 0.05). There was statistically significant main effect of time on isomeric strength UT: (*p* = 0.008), ES = 0.65; MTr: (*p* = 0.007), ES = 0.80; LT: (*p* = 0.009), ES = 0.48; SA:  (*p* = 0.002)ES = 0.93; Supr: (*p* = 0.005), ES = 0.85; A, D: (*p* = 0.003), ES = 0.90; L.D: (*p* = 0.002), ES = 0.93 (Table 5).

There were statistically significant group effects on isometric strength UT: (*p* = 0.004), ES = 0.39; MTr: (*p* = 0.009), ES = 0.16; LT: (*p* = 0.005), ES = 0.38; SA: (*p* = 0.006), ES = 0.36; Supr.: (*p* = 0.004), ES = 0.39; A.D: (*p* = 0.003), ES = 0.40; LD: (*p* = 0.007), ES = 0.33 (Table 5).

There were statistically significant interaction effects (time × group) on isometric strength UT: (*p* = 0.007), ES = 0.62; MTr: (*p* = 0.006), ES = 0.77; LT MA: (*p* = 0.008), ES = 0.45; SA: (*p* = 0.002), ES = 0.91, Supr.: (*p* = 0.004), ES = 0.84; A. D: (*p* = 0.003), ES = 0.88; LD: (*p* = 0.002), ES = 0.92 (Table 5).

## 4. Discussion

In this study, we compared the effect of two different protocols on the quantum of isometric improvement in overhead athletes with SIS. This study has tried to fill in the void due to the dearth of evidence in the area of athletic injuries. The primary findings of this study indicate that the largest improvement in terms of isometric strength for between-groups analysis (descending order) occurred in AD, Supr, and UT muscles in the PRE plus MT group. The isometric strength of A.D and UT decreased while that of Supr. increased as noted in the between-group analysis. Although lesser, the MCE group also reported significant improvement for all muscles except for Supr. For the within-group effects, the highest improvement was found for Supr and SA muscles in the PRE plus MT group.

The results of the study demonstrated that both groups showed significant large improvement with respect to time for isometric strength of SA and LD muscle. Results also showed that there was a significant small increase or optimization in isometric strength for A.D and UT, Supr. muscle for group effects. The interaction effects (group × time) were largest for LD and SA muscles, respectively. In terms of effect size interpretation, the changes were large for time and interaction effects, while for group effects, it was small [33]. The MCE group showed significant improvement in all examined muscles except for Supr. at both time points. The results demonstrated that both interventions were effective in improving and optimizing isometric strength; nevertheless, athletes who underwent combined PRE plus MT presented a more pronounced increase or optimization of isometric strength.

The increase in isometric strength from the present study is in line with other research findings involving therapeutic exercises [15, 35]. The abovementioned results are in synchrony with the study performed to evaluate isometric strength changes in shoulder impingement syndrome after two different exercise interventions in female subjects with shoulder impingement syndrome [35]. The isometric strength in this study was measured by the isokinetic dynamometer. The percentage change for the isometric strength for MT, LT, SA, and Supraspinatus was 53.01, 50.74%, 36.45%, and 58.01% (*p* < 0.001), respectively, for the experimental group. On the contrary, the percentage change was found nonsignificant in the control group (*p* > 0.05) [35]. In another study, the composite isometric strength measurement was reported to have improved by 19.24% in the manual therapy plus exercise group and by 6.55% in the exercise therapy group [15]. The percentage changes in the current study are also on similar lines (increasing trend) to the abovementioned studies for isometric strength of UT, MTr., LT, SA, supraspinatus, A.D, and LD (24.05%, -21.48%, -22.96%, -41.78%, -33.43%, 22.13%, and -37.22%, respectively). The slightly higher isometric strength values in the abovementioned study could have been due to proper stabilization and support provided by the isokinetic device [35]. Although significant, the percentage change was much lesser in the MCE group in the present study. Concerning the other study, the gains might have been lesser than our study possibly due to the heterogeneous population (age group) and shorter duration of the intervention (3 weeks) [15]. In another study, which evaluated the effectiveness of conservative therapy versus motor control and strengthening exercises, a total of 35 subjects were subjected to 4 weeks (3 sessions per week) of treatment in both groups. The authors reported that there was a significant improvement in the external and internal rotator strength in the motor control and strengthening groups [36]. The combination of progressive resistance exercise and manual therapy is a promising field of research in SIS rehabilitation. A study that compared the effects of exercise therapy with and without manual therapy found that fifty-two subjects of SIS submitted to six sessions in the experimental group (exercise and manual therapy) had significantly higher isometric muscle strength after an intervention of 3 weeks [15]. In continuation, thirty-three subjects with SIS were submitted to similar intervention for 6 weeks, similar improvement was observed in the strength and the function measures outcomes [24].

In contrast, few studies have reported contrary findings than that of the present study. In one of the studies, forty-six patients were randomly divided into two groups, i.e., exercise plus manual therapy and exercise therapy alone; both the groups received 4 weeks of intervention and concluded that the addition of manual therapy to an exercise protocol did not enhance muscle strength of individuals having SIS [37]. Minor to inconclusive evidence on strength improvement has been concluded by few meta-analytic studies for integrated exercise and manual therapy programs [22, 23, 38]. Mainly, these reviews have concluded that most of the studies to date lack strong methodology, homogenous group, and amalgamated protocol for effective management.

The present study was a randomized controlled trial with a homogenous sample having SIS dysfunction and the protocol is a broad spectrum, based on best available evidence and encompasses progressive resistance exercise along with stretching exercises, manual therapy which covers up for the lacunae of previous researches and future recommendations of meta-analysis. There are three plausible reasons for the results obtained in the present study.

Firstly, the combined exercise therapy and manual therapy protocol increased the activity of the scapular muscles by initially inhibiting the overactive muscles like Ant. Deltoid and UT as reported in literature [39]. These two overactive muscles are known to disturb the functioning of force couples around the shoulder complex. The UT and SA force couple during the 0° to 30° of movement is impaired in SIS. As per the Janda approach, due to pain, all periscapular muscles undergo inhibition of their activity except the antigravity muscle like the upper trapezius [40]. This leads to overactivity of the upper trapezius, and it disturbs the strength ratio between UT and SA. In continuation, when the subject takes the arms overhead, at that time, the two force couples of UT/LT and UT/SA are physiologically active. However, due to SIS, this force couple is again disrupted, and LT and SA are inhibited and UT becomes overactive [40, 41]. The protocol used in this study might normalize these force couple by first inhibiting the overtly active UT activity and then strengthening the other scapular muscles like LT and SA [42, 43]. The stretching exercise component of the protocol alters the muscle spindle (Ia and II afferents) and perhaps the Golgi tendon organ (Ib afferents) output to the central nervous system. Such an altered afferent drive is supposed to downregulate the activity of the *α*-motor neurons and reduce the overactivity of the muscles [43]. Secondly, due to the chronicity of SIS, there is an insidious decrease in rotator cuff muscle activity and functioning. This usually starts with the supraspinatus muscle, and if the dysfunction is not addressed effectively, the other remaining rotator cuff muscles also shut down, and there is a reduction in abduction force. Rotator cuff muscles are also called the core muscles of the shoulder joint; in the presence of weak core muscles, the superficially placed global muscles like anterior deltoid start overworking.

Reverse scapulohumeral rhythm is one of the results of this muscular dysfunction. In the present study, there was a reduction in the overactivity of the anterior deltoid and an increase in activity of the supraspinatus muscle strength which suggests that this protocol of combined PRE plus MT has the potential to correct this imbalance [42]. Thirdly, one of the prime findings in SIS is a large reduction in external rotation force, and it is also a vital prognostic marker. The usual postural phenotype of the scapular position reported and seen in SIS is excessive anterior tipping, internal rotation, and scapular abduction. Due to this posture, the patients with SIS have rounded and stooped shoulders that further increase the probability of impingent during overhead activities [9, 44]. Glenohumeral external rotation of the shoulder requires a stable preposition and kinematics of scapular retraction, adduction, and scapular external rotation [41, 45]. The combination of PRE plus MT in the present study increased the isometric muscle strength of MT, LT, and LD that might normalize the kinematics of scapula and muscle length-tension relationship for optimal shoulder external rotation force generation.

The strength of the study lies with the fact that the present study is amongst the few studies that have examined the combined effects of PRE plus MT and compared it with another effective treatment protocol of MCE in overhead athletes with SIS. Secondly, the protocol which has been adopted is an amalgamated protocol from best practice patterns reported by authors in research and no adverse effects were reported. Thirdly, the manual therapy techniques used in the study was performed by practitioner having requisite experience after postgraduate education.

### 4.1. Limitations

In the present study, only male overhead athletes were recruited, which could have a bearing on the generalization of the results of the study for all overhead athletes. Secondly, there was no follow-up with the athletes after the intervention.

The compliance of the athletes with the treatment protocols in both groups was good. There were no adverse effects reported by any of the athletes during the treatment period. The dropout rate was 9.09% (which was expected for the study of this duration). Future studies are indicated to investigate the long-term effects of combined progressive resistance exercise and manual therapy in athletes.

## 5. Conclusion

The study reveals that the PRE plus MT is a better option compared to MCE in optimizing and improving isometric scapulothoracic muscle strength in overhead athletes. Between groups, the analysis found the largest isometric strength improvement in PRE plus MT group for A.D, followed by Supr. and UT muscles. Supr. isometric muscle strength increased while that of A.D and UT decreased. Our study has led us to conclude that PRE plus MT intervention is more effective and clinically superior compared to MCE intervention for inducing improvement in the isometric strength of SIS overhead athletes.

## Figures and Tables

**Table 1:** Positioning of athletes for isometric strength measurement of muscles.

Muscle	Procedure
UT	With the subject in high sitting, the dynamometer was pushed down over the suprascapular fossa and the subject was told to perform shoulder elevation
MTr.	After placing the dynamometer on the mid-way point of the spine of the scapula and acromion, the dynamometer was pushed laterally while the subject was asked to keep the arm in an abducted externally rotated position while the subject lay prone.
LT	After placing the dynamometer on the mid-point of the acromion and medial root spine of the scapula, the dynamometer was pushed in superolateral direction while the subject was prone with arm in 140° of flexion.
Supr.	The subject was in high sitting and the shoulder is put in 30 degrees of abduction and in 90 degrees of flexion and counterforce was given just proximal to the elbow.
S.A	After placing the dynamometer on the forearm ulnar border while the shoulder and elbow were flexed to 90°, the subjects were asked to lift the point of the elbow towards the ceiling for which the counterforce was applied.
A.D.	After placing the dynamometer just proximal to the elbow, the subject in high sitting was asked to counter the downward force applied by the examiner while the shoulder was in 90° of flexion.
L.D	After placing the dynamometer on the posterior aspect of the elbow, the subject in the seated position was asked to counter the anterior direction while the shoulder was in 30° extension and 90°elbow flexion.

Abbreviation: UT: upper trapezius; MTr.: middle trapezius; LT: lower trapezius; SA: serratus anterior; Supr: supraspinatus; A.D: anterior deltoid; LD: latissimus dorsi.

**Table 2:** Detailed description of the intervention in the PRE plus MT group.

	Progression (repetition and sets)	
	First 4 weeks	Next 4 weeks
Phase 1		
Range of motion exercises Stretching(7 days a week)	10 × 1 (daily) 5 times ×30-sec hold	10 × 1 (daily) 5 times ×30-sec hold
Manual therapy		
Thoracic PA glides in prone Post. and inf. glide GH joint.	6 sessions	6 sessions
Strengthening exercises		
PRE shoulder IR and ER(NP) PRE shoulder extension PRE scap. retr and protraction(supine) Scapular retraction with tuck-in chin	1^st^: 10 reps × 2 sets 2^nd^: 10 reps × 3 sets (grade-up resistance band to red to green to blue)	na
Phase 2		
Shoulder elevation and flexion (up to 90°) and resisted extension Shoulder ER and IR [45°-90°] Quadruped push-up plus “camel” Scapular “T” and “Y” exercise	3^rd^ week 10 reps × 2 sets 4^th^ week 10 reps × 3sets (grade up from yellow to red)	2 − 3 sets × 10 reps (progressing from green to blue)
Phase 3		
(In addition to phase 2 exercises) Chair press Protraction-plank	na	5th to 8th week - 2 -3sets x 10 reps

Abbreviation: PA: posteroanterior; reps: repetitions; PRE: progressive resistance exercise; na: not applicable; Post: posterior; Inf: inferior; NP: neutral position; scap: scapula; retr: retraction.

**Table 3:** Detailed description of the intervention in the MCE group.

	Progression (repetition and sets)	
	First 4 weeks	Next 4 weeks
Phase 1		
Range of motion exercises		
Frontal pl. abd.	20 times × daily	20 times × daily
Neck retr.	20 times × daily	20 times × daily
Stretching exercise		
UT	6 times × 20-sec hold	6 times × 20-sec hold
Phase 2		
Range of motion exercises		
Shoulder shrugging	20 times × daily	20 times × daily
Shoulder retr.	20 times × daily	20 times × daily
Stretching exercise		
Pec. major	6 times × 20-sec hold	6 times × 20-sec hold

Abbreviations: MCE: motor control exercise; pl.: plane; abd: abduction; retr: retraction; UT: upper trapezius; Pec. major: pectoralis major.

**Table 4:** Comparison of the demographic characteristics and outcome measures between groups at baseline.

Characteristics	PRE plus MT group (*n* = 40) $\text{Mean }\underset{¯}{\pm }\text{ SD}$	MCE group (*n* = 40) Mean ± SD	*p*
Age (years)	21.30 ± 2.10	21.80 ± 2.80	0.93
Height (cm)	178 ± 2.50	177 ± 2.30	0.76
Weight (kg)	72.3 ± 1.20	71.2 ± 1.30	0.66
Body mass index (BMI) (kg/m^2^)	22.80 ± 0.68	22.57 ± 0.72	0.40
Symptom duration (months)	5.50 ± 2.20	4.42 ± 1.88	0.32
VAS level (E.A)	6.52 ± 0.35	6.14 ± 0.18	0.72
Years of playing	4.50 ± 1.50	4.30 ± 1.30	0.42
UT Str. (N)	234.02 ± 19.15	233.55 ± 21.13	0.98
MTr. Str. (N)	189.84 ± 22.65	190.76 ± 22.59	0.86
LT Str. (N)	197.64 ± 18.64	189.01 ± 20.36	0.16
SA Str. (N)	117.91 ± 10.07	121.10 ± 11.02	0.55
Supr. Str. (N)	143.91 ± 16.74	142.08 ± 21.11	0.41
A.D. Str. (N)	212.48 ± 13.05	212.03 ± 12.43	0.44
LD Str. (N)	119.74 ± 12.86	121.88 ± 9.70	0.08

Abbreviations: BMI: body mass index; PRE plus MT group: progressive resistance exercises plus manual therapy group; MCE group: motor control exercise group; UT: upper trapezius; MTr.: middle trapezius; LT: lower trapezius; SA: serratus anterior; Supr: supraspinatus; AD: anterior deltoid; LD: latissimus dorsi; Str: strength; VAS: visual analogue scale; E.A: elevation activity; *p*: level of significance set at <0.05; ^∗^level of significance *p* < 0.05; N: newtons.

**Table 5:** Mean change ± SD of outcome measure of both group and summary of mixed model ANOVA.

Variables	Time (wk)	M.D. (G)		T		G		G∗T	
		PRE plus MT (n:40)	MCE (n:40)	p	E.S	p	E.S	p	E.S
UT Str.	4^th^	23.35 ± 21.21^∗^	0.82 ± 1.27^∗^	0.008^∗^	0.65	0.004^∗^	0.39	0.007^∗^	0.62
	8^th^	36.26 ± 26.97^∗^	2.02 ± 1.84^∗^						
MT Str.	4^th^	−20.57 ± 14.66^∗^	−0.34 ± 1.65^∗^	0.007^∗^	0.80	0.009^∗^	0.16	0.006^∗^	0.77
	8^th^	−40.78 ± 13.29^∗^	−1.95 ± 2.02^∗^						
LT Str.	4^th^	−25.02 ± 23.34^∗^	−0.94 ± 1.43^∗^	0.009^∗^	0.48	0.005^∗^	0.38	0.008^∗^	0.45
	8^th^	−45.39 ± 29.71^∗^	−1.38 ± 3.10^∗^						
SA Str.	4^th^	−14.02 ± 7.96^∗^	−0.54 ± 0.54^∗^	0.002^∗^	0.93	0.006^∗^	0.36	0.002^∗^	0.91
	8^th^	−49.27 ± 9.29^∗^	−2.52 ± 1.47^∗^						
Supr. Str.	4^th^ 8^th^	−31.77 ± 11.96^∗^ −55.32 ± 14.10^∗^	−0.88 ± 1.47^∗∗^ −0.61 ± 5.07^∗∗^	0.005^∗^	0.85	0.004^∗^	0.39	0.004^∗^	0.84
A.D Str.	4^th^ 8^th^	21.03 ± 7.96^∗^ 47.03 ± 10.58^∗^	1.33 ± 1.02^∗^ 2.24 ± 1.43^∗^	0.003^∗^	0.90	0.003^∗^	0.40	0.003^∗^	0.88
LD Str.	4^th^ 8^th^	−14.27 ± 9.51^∗^ −44.57 ± 7.59^∗^	−0.38 ± 0.91^∗^ −1.53 ± 1.26^∗^	0.002^∗^	0.93	0.007^∗^	0.33	0.002^∗^	0.92

Abbreviation: PRE plus MT group: progressive resistance exercise plus manual therapy group; MCE group: motor control exercise group; *n*: number of athletes; UT: upper trapezius; MTr: middle trapezius; LT: lower trapezius; SA: serratus anterior; Supr: supraspinatus; A.D: anterior deltoid; LD: latissimus dorsi; time (wk): measurement at 4^th^ and 8^th^ week; M.D (G).: mean change groups; E.S: effect size: time effects; G: group effects; G∗T: group × time interactions; Str: strength; ^∗^: significant at *p* < 0.05; ^∗∗^: nonsignificant at *p* > 0.05.

## Data Availability

The statistical clinical data used to support the findings of this study are included within the article.

## Abbreviations

SIS: Shoulder impingement syndrome
HHD: Hand held dynamometer
UT: Upper trapezius
MTr.: Middle trapezius
LT: Lower trapezius
SA: Serratus anterior
A.D: Anterior deltoid
Supr: Supraspinatus
LD: Latissimus dorsi.
